# Gelsolin amyloidosis presenting with nephrotic syndrome: a case report and molecular insights

**DOI:** 10.3389/fmed.2026.1798985

**Published:** 2026-05-18

**Authors:** Silin Xiang, Peng Bi, Dazhou Chen, Wen Tang, Yuan Li, Yali Zhou, Dedong Kang, Chuqi Pan, Feng Wan, Jin Yu, Xuanli Tang

**Affiliations:** 1 Department of Nephrology, Hangzhou TCM Hospital Affiliated to Zhejiang Chinese Medical University, Hangzhou, China; 2 Department of Pathology, Sir Run Run Shaw Hospital, School of Medicine, Zhejiang University, Hangzhou, China; 3 Department of Nephrology, The First Affiliated Hospital of Zhengzhou University, Zhengzhou, China; 4 Department of Anatomy, Showa Medical University School of Medicine, Tokyo, Japan

**Keywords:** gelsolin, gene mutation, hereditary amyloidosis, immunohistochemistry, mass spectrometry, nephrotic syndrome

## Abstract

Familial Amyloidosis of Finnish type (FAF) is a rare autosomal dominant hereditary amyloidosis associated with genetic variants of gelsolin. This condition is characterized by ophthalmologic abnormalities, progressive cranial neuropathy, and cutis laxa, while renal impairment is rare. We report a gelsolin amyloidosis in a 58-year-old man with nephrotic syndrome and slowly progressive kidney dysfunction, associated with a gelsolin gene mutation (c.480C > A, p.Asn160Lys). Initial renal biopsy showed segmental IgA deposition, moderate mesangial expansion with weak PAS positivity, and parallel bundles of 10 nm fibrils, but only trace Congo red staining. A second biopsy 2 years later revealed IgA-dominant deposition, nodular sclerosis, similar fibrils, glomerular basement membrane lamination, and weakly positive Congo red. The diagnosis of gelsolin amyloidosis was confirmed by mass spectrometry and immunohistochemistry. Clinically, the patient had no neuropathy or family renal disease but presented with gastrointestinal symptoms, and endoscopy confirmed vascular amyloid deposition. Molecular structure analysis suggested that the mutation may loosen the protein structure while preserving its calcium-binding ability. Combining with the literature, we discuss the clinicopathological features and predicted protein functions of mutated gelsolin, aiming to elucidate its pathogenic mechanisms and deepen the understanding of hereditary amyloidosis.

## Introduction

Familial Amyloidosis of Finnish type (FAF), also known as hereditary gelsolin amyloidosis, is a rare autosomal dominant hereditary amyloidosis caused by mutations in the gelsolin (GSN) gene located on chromosome 9q33.2 (1). The symptoms of FAF are multifaceted, most commonly including cranial neuropathy, corneal lattice dystrophy, distal sensorimotor neuropathy, and dermatological changes (2). Rare cases presenting with proteinuria, nephrotic syndrome (NS), and end-stage renal failure have been reported (3). Here we report a case of chronic kidney disease (CKD) caused by a gelsolin gene mutation (p.Asn160Lys). The patient presented with NS, gastrointestinal involvement, but without neuropathy or corneal lattice dystrophy.

## Case presentation

A 58-year-old Chinese man was admitted in January 2025 for the evaluation of persistent urinary abnormalities documented for over 30 years and elevated serum creatinine (Scr) observed for more than 2 years. Initial urinalysis three decades ago showed urine protein (UP) levels fluctuating between trace and 1 + without microscopic hematuria. Two years before admission, urinalysis demonstrated UP 2 + and an elevated Scr level of 120 μmol/L. His medical history was notable for hypertension (HTN) for 12 years (with peak blood pressure >160/100 mmHg), chronic non-atrophic gastritis, reflux esophagitis (both for 3 years), and a 35-year smoking history (averaging 20 cigarettes per day). No family history of known renal disease. The patient was taking sacubitril/valsartan (100 mg once daily) and levamlodipine besylate (2.5 mg once daily) for blood pressure control, along with omeprazole for gastroprotection.

## First renal biopsy

**Figure 1:**
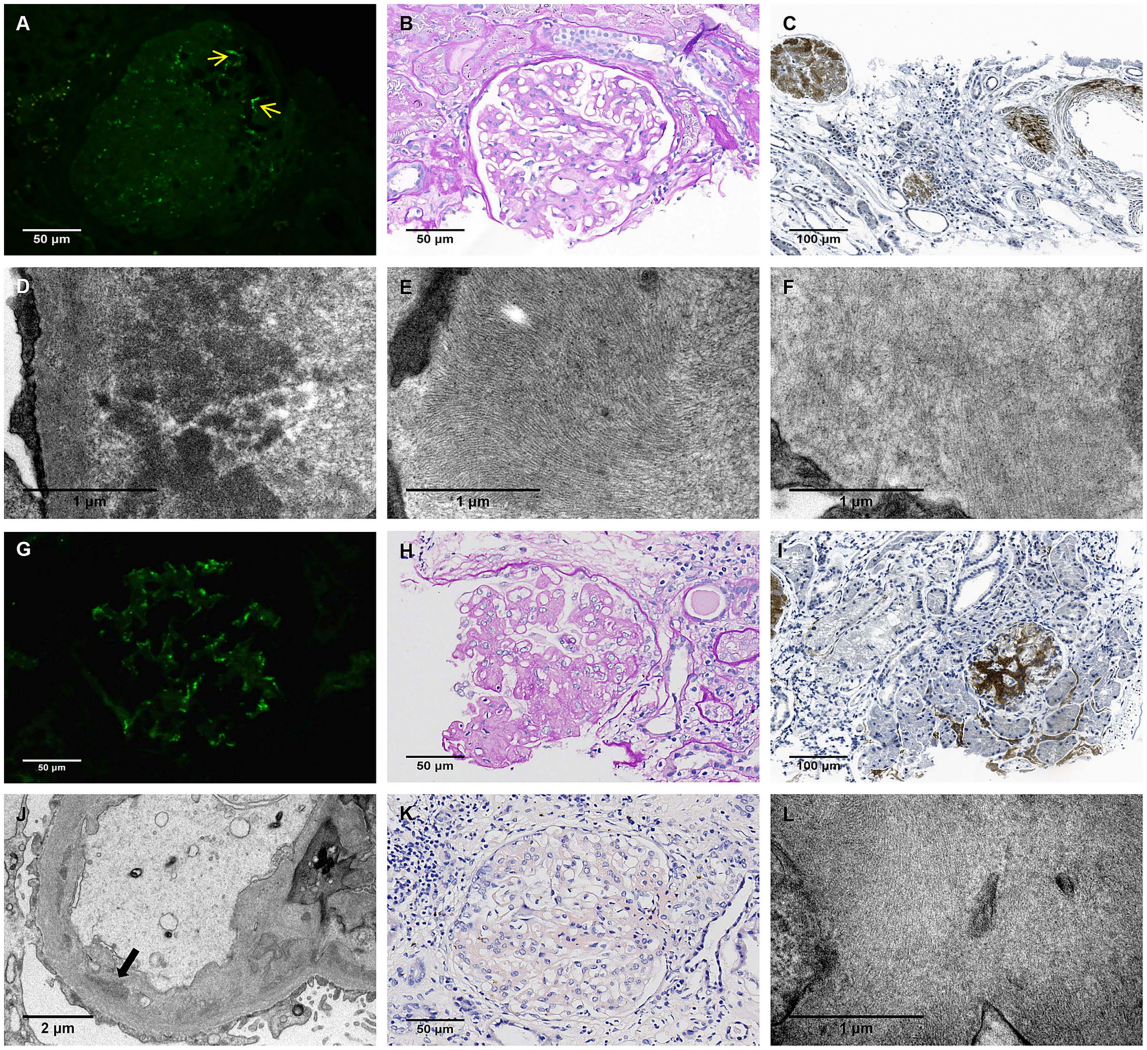
Pathological findings of the first and second renal biopsy. **(A–F)**: First renal biopsy. **(A)** Segmental IgA deposition in the mesangial area (yellow arrow, IF, 400X). **(B)** Mesangial expansion with deposition of weakly PAS-positive material (PAS, 400X). **(C)** Strong gelsolin deposition in glomeruli, interstitium, and vasculature (IHC, 200X). **(D)** Amorphous dense deposits in the mesangium area (EM, 30000X). **(E)** Long, parallel, curved fibrils (10 nm in diameter) within the mesangium, arranged in bundles (EM, 30000X). **(F)** Short, rigid, randomly oriented fibrils (10 nm in diameter) in the subepithelial space (EM, 30000X). **(G–L)**: Second renal biopsy. **(G)** Diffuse mesangial deposition of IgA (IF, 400X). **(H)** Nodular sclerosis with weak PAS positivity (PAS, 400X). **(I)** Prominent gelsolin deposition in glomeruli and the interstitium (IHC, 200X). **(J)** Subendothelial and mesangial amorphous dense deposits (black arrow, EM, 10000X). **(K)** Weakly positive Congo red staining was mainly observed in the mesangial area of the glomeruli (Congo red staining, 400X). **(L)** Randomly oriented, non-branching fibrils (10 nm in diameter) within the mesangial area, lacking the parallel arrangement seen in the first biopsy (EM, 30000X). IF, immunofluorescence; PAS, Periodic Acid-Schiff stain; IHC, immunohistochemistry; EM, electron microscope.

A renal biopsy performed externally in August 2023 revealed ambiguous pathology. Immunofluorescence (IF) showed segmental glomerular deposits of all immunoglobulins (Igs), complements, and light chains, with predominant IgA (Figure 1A). Light microscopy (LM) of 27 glomeruli identified glomerulosclerosis (8/13 globally, 5/13 segmentally), with mesangial hypercellularity, matrix expansion containing amorphous PAS-positive material, moderate interstitial fibrosis and tubular atrophy (IFTA), and arteriolar sclerosis (Figure 1B). Congo red staining was equivocal, and DNAJB9/fibronectin immunohistochemistry (IHC) was negative, ruling out specific glomerulopathies such as fibrillary glomerulonephritis and fibronectin glomerulopathy. Electron microscopy (EM) revealed mesangial expansion with abundant short, rigid rod-shaped fibrils and parallel 10 nm filaments, which were also present in subendothelial and subepithelial spaces, along with amorphous electron-dense deposits (EDDs) in the mesangium area and diffuse foot process effacement (Figures 1D–F). These combined morphological features did not yield a definitive pathological diagnosis.

## Treatment and follow-up

A comprehensive workup for secondary causes (monoclonal Ig, cryoglobulins, malignancy, autoimmune diseases) was negative. The patient was started on prednisone 20 mg daily, hydroxychloroquine, dapagliflozin, and irbesartan, but showed no improvement. Kidney function instead progressed slowly (24hUP 6.6 g/day, albumin (ALB) 29.9 g/L, Scr 145 μmol/L). Immunosuppression was then switched to *Tripterygium wilfordii*, yet NS persisted, with a slight decline in renal function, leading to re-admission in January 2025 for further evaluation and possible change in immunosuppressive therapy.

## Second renal biopsy

IF showed diffuse mesangial IgA, C3, and light chains (1–2+), with other Igs/complements minimal (Figure 1G). LM revealed severe glomerulosclerosis (8/10 globally, 1/10 segmentally), marked mesangial expansion with nodular amorphous PAS-weak deposits, severe IFTA, and arteriosclerosis (Figure 1H). EM displayed thickened laminated glomerular basement membrane (GBM) with amorphous EDDs and random 10 nm fibrils, but lacked the parallel filament bundles seen in the first biopsy (Figures 1J,L). Congo red was weakly positive with equivocal birefringence (Figure 1K); DNAJB9/fibronectin were negative. For definitive diagnosis, laser microdissection with mass spectrometry (LMD/MS) identified high spectral counts for amyloidosis-associated proteins (apolipoprotein E, serum amyloid P-component) and gelsolin, alongside Igs/complements, indicating hereditary gelsolin amyloidosis and suspected IgA nephropathy (IgAN). IHC confirmed strong gelsolin deposition in the glomeruli, interstitium, and vessels in both biopsies (Figures 1C,I).

## Treatment adjustment and follow-up

Immunosuppressive therapy was initiated in January 2025 in consideration of superimposed IgAN, comprising prednisone (40 mg daily) and cyclophosphamide; the cumulative dose of cyclophosphamide reached 2.8 g by the end of April. Treatment was suspended in May due to the development of a lung abscess; after the infection was controlled, the regimen was adjusted to low-dose prednisone (15 mg/d). No renal response was observed; however, renal function remained stable.

**Figure 2:**
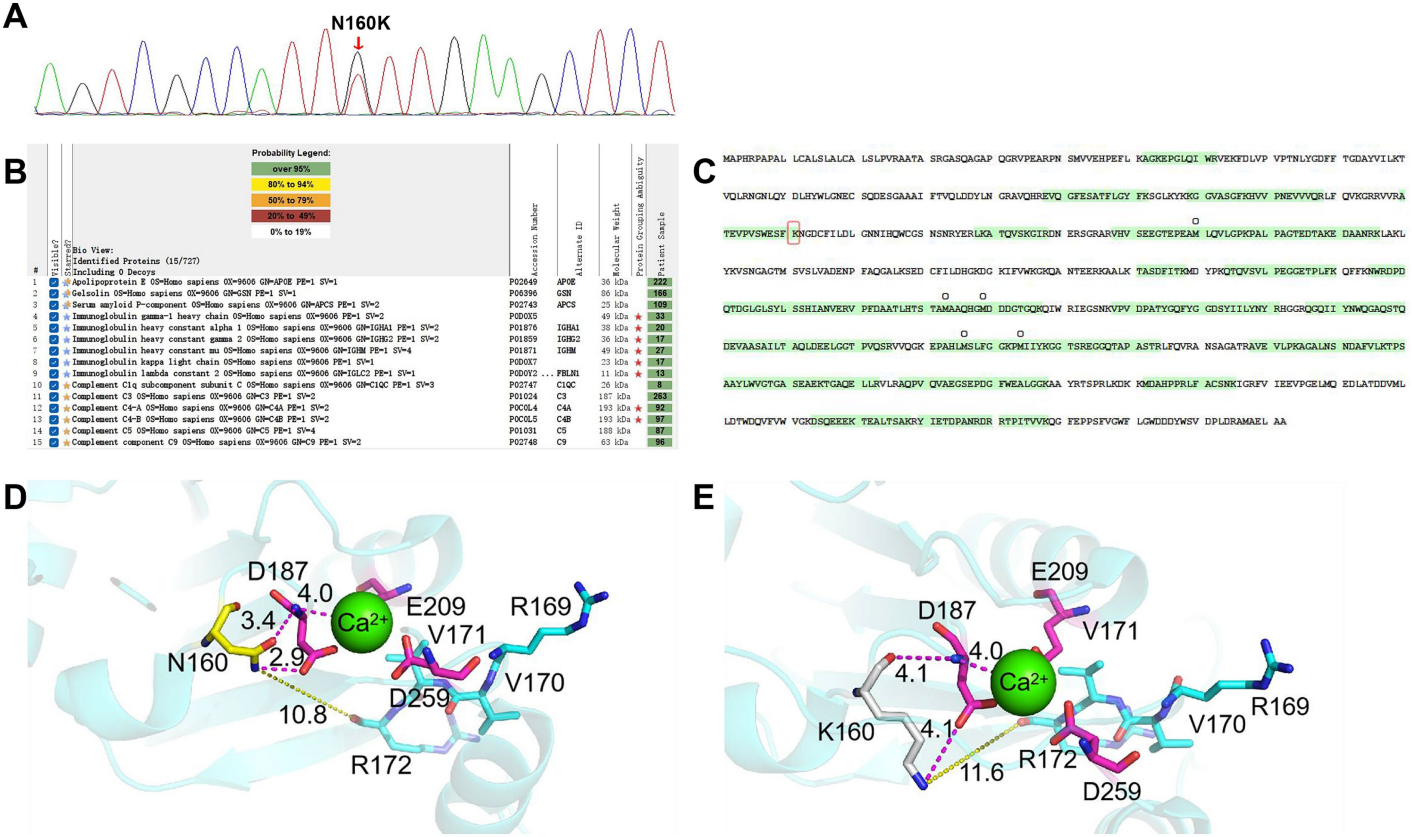
DNA sequencing, proteomic identification, and structural analysis of gelsolin. **(A)** Heterozygous mutation site in the GSN gene (c.480C > A). **(B)** High spectral counts for amyloidosis-associated proteins and gelsolin, alongside Igs/complements, were detected in renal tissue. **(C)** Coverage of the gelsolin amino acid sequence with the detected portions highlighted in green and the mutation site marked by a red box. **(D)** Structure of normal gelsolin. The furin domain and key activity residues were shown in a stick model. The functional domain (red), a calcium ion (green), the side domain (N160, yellow), and the furin cleavage domain (blue) are shown. Inter-domain distances are indicated by dashed lines with values in Ångströms (Å). **(E)** Structure of gelsolin with a K160 mutation. The mutated side domain is shown in grey, with the other domains presented for comparison (see panel **D**).

Follow-up examinations were negative for neurological, corneal, or cutaneous involvement. However, Congo red-positive amyloid deposits were identified in gastric (2023) and intestinal (2025) arterioles on endoscopic biopsies. Genetic testing revealed a likely pathogenic heterozygous GSN mutation (c.480C > A, p.Asn160Lys), absent in the patient’s son (Figure 2A). Comprehensive genetic analysis of the patient’s parents was not feasible, as both were deceased. MS analysis corroborated these findings by detecting peptide fragments specific to the mutant allele in the patient, while no corresponding wild-type peptides were identified (Figures 2B,C). Furthermore, molecular structure analysis was used to predict the impact of the mutation on the interaction between the functional domain and the calcium ion. The crystal structure (pdb entry:3FFK) of gelsolin was loaded from the RCSB Protein Data Bank.^1^ The mutation at key residue N160K was executed in PyMol 2.3.0a.^2^ PyMol was used to visualize and analyze interactions between the furin domain and the key residue. In the wild-type structure, the side chain of N160 forms a close interaction with D187 in domain 2, stabilizing the conformation of the calcium-binding pocket (Figure 2D). Upon mutation to K160, this interaction is attenuated, as reflected by an increased distance (from 2.9 Å to 4.1 Å), thereby loosening the key protein architecture. However, the mutation did not significantly alter the distance between D187 and the calcium ion (~4.0 Å) (Figure 2E).

Based on the synthesized clinical, pathological, and molecular evidence, we confirm a diagnosis of FAF with renal involvement induced by a GSN gene mutation.

## Discussion

Gelsolin is a cytosolic calcium–sensitive protein that was first shown to regulate actin gel–sol transformation in macrophages (4). The binding of gelsolin to actin regulates the transition of actin from a fibrillary to a soluble form that is required for macrophage movement (5). Gelsolin amyloidosis is a rare autosomal dominant hereditary amyloidosis. The most common G654A/T mutations impair calcium ion binding in gelsolin domain 2, leading to misfolding and aberrant furin cleavage in the Golgi. This generates a C-terminal 68 kDa fragment (C68), which is further processed extracellularly into 8- and 5-kDa amyloidogenic fragments. These fragments cause systemic deposition, manifesting as cranial neuropathy, corneal lattice dystrophy, cutis laxa, and other symptoms (6, 7). However, follow-up examinations revealed no neurological, corneal, or cutaneous abnormalities in our patient. The molecular structure analysis suggests that the N160K mutation may destabilize the key protein architecture, promoting furin-mediated cleavage and subsequent amyloid fibril formation, which is consistent with previous studies (3, 8). Nevertheless, the interaction between domain 2 and the calcium ion is preserved, which could explain the absence of typical neurological symptoms over the long disease course. In addition, a similar case of the Asp163Asn mutation presented with obvious kidney injury but no other organ involvement (9). The researchers suspect that variations at different loci of the GSN gene contribute to phenotypic differences. Besides, parental evaluation was not possible in our study, suggesting either a *de novo* event or incomplete penetrance. Taken together, those observations highlight variability in clinical expression and the importance of genetic counseling. Notably, the patient exhibited gastrointestinal involvement, characterized by vascular amyloid deposition. We speculate that such vascular deposition may predispose to multi-system involvement—a pattern often seen in AL amyloidosis—although this specific presentation has not been previously reported for FAF in the literature (10, 11).

Gelsolin amyloidosis rarely presents with clinically significant renal involvement (2). This low frequency is further illustrated by the study by Nikoskinen et al. (12), in which renal impairment was observed in only 3% of a 227-patient Finnish cohort with gelsolin amyloidosis, underscoring the exceptional nature of renal presentation in this disease (4). Renal involvement in gelsolin amyloidosis (AGel) typically manifests in older patients, who frequently present with comorbidities such as HTN due to no vessel involvement and diabetes. Notably, the disease exhibits a more indolent clinical course and a more favorable prognosis compared to other forms of systemic amyloidosis. Our patient presented with NS, HTN, and renal involvement. Distinctively, the renal pathology included mild subepithelial amyloid deposition—a feature not previously reported in gelsolin amyloidosis (2). This process, in turn, may amplify amyloid fibril production and enhance fibril invasiveness. This could explain the widespread vascular deposition, multi-organ involvement, and GBM lamination observed, likely resulting from tissue remodeling.

In addition, a subset of cases (3/12) shows concurrent immunoglobulin deposits, a feature that may lead to misdiagnosis as fibrillary glomerulonephritis (FGN) (13). However, the pathological features of this case are distinctly different from those of classic FGN. IF revealed predominantly mesangial deposition of IgA and C3, rather than the IgG-dominant pattern typical of FGN. Additionally, EM revealed amyloid fibrils measuring 10 nm in diameter, which were thinner than those in FGN, with some also lacking typical rigidity—a finding that necessitates confirmation by MS. The pathogenesis of immunoglobulin deposits remains uncertain. In our patient, the diffuse mesangial deposition of IgA and C3—later confirmed by MS—together with mesangial hypercellularity, is most consistent with concurrent IgAN, although a contribution from gelsolin-associated antibodies or non-specific trapping cannot be completely ruled out. Our decision to initiate immunosuppressive therapy was primarily based on the pathological findings suggested by concurrent IgAN, although it remained unclear whether IgAN was the predominant driver of renal injury. However, the patient showed no clinical response to either initial or intensified immunosuppression, which does not support superimposed immune complex-mediated glomerulonephritis; therefore, we shifted our management strategy toward supportive care aimed at slowing disease progression—including strict blood pressure control, avoidance of nephrotoxic agents, regular monitoring of nephrocardiovascular parameters, and patient education. Accordingly, clinical management should focus on controlling the complications of amyloidosis and delaying the progression of renal failure.

In summary, this case highlights atypical gelsolin amyloidosis with predominant renal and systemic arteriolar involvement, without the typical family history or cutaneous, neurological, and ocular manifestations. Pathological findings could mimic fibrillary glomerulonephritis or other inherited nephropathies. This case underscores the risk of misdiagnosis due to its atypical features and highlights the critical role of MS and genetic testing in diagnosis, complemented by molecular structure analysis to elucidate the mutation’s functional impact.

## Patient perspective

The patient was initially concerned about the prolonged diagnostic uncertainty. After the diagnosis of hereditary amyloidosis was confirmed, he felt relieved to finally understand the cause of his long-standing proteinuria. He actively participated in treatment decisions and adhered to both immunosuppressive (for suspected IgAN) and supportive therapies. Despite no renal response to immunosuppression, he remained compliant with follow-up and appreciated the multidisciplinary care provided.

## Data Availability

The datasets presented in this study can be found in online repositories. The names of the repository/repositories and accession number(s) can be found at: https://ngdc.cncb.ac.cn/gsa-human, HRA015853.

## Footnotes

1
https://www.rcsb.org/

2
http://www.pymol.org
